# Crystal Structure and Pyridoxal 5-Phosphate Binding Property of Lysine Decarboxylase from *Selenomonas ruminantium*

**DOI:** 10.1371/journal.pone.0166667

**Published:** 2016-11-18

**Authors:** Hye-Young Sagong, Hyeoncheol Francis Son, Sunghwan Kim, Yong-Hwan Kim, Il-Kwon Kim, Kyung-Jin Kim

**Affiliations:** 1 School of Life Sciences, KNU Creative BioResearch Group, Kyungpook National University, Daegu, 702–701, Republic of Korea; 2 New Drug Development Center, Daegu-Gyungpook Medical Innovation Foundation, Daegu, 701–310, Republic of Korea; 3 School of Nano-Bioscience and Chemical Engineering, Ulsan National Institute of Science and Technology (UNIST), Ulsan, 689–798, Republic of Korea; 4 Biopoecess Research Depart. R&D Center, DAESANG Corp., Icheon-si, Gyeonggi-do, 17384, Republic of Korea; Russian Academy of Medical Sciences, RUSSIAN FEDERATION

## Abstract

Lysine decarboxylase (LDC) is a crucial enzyme for acid stress resistance and is also utilized for the biosynthesis of cadaverine, a promising building block for bio-based polyamides. We determined the crystal structure of LDC from *Selenomonas ruminantium* (*Sr*LDC). *Sr*LDC functions as a dimer and each monomer consists of two distinct domains; a PLP-binding barrel domain and a sheet domain. We also determined the structure of *Sr*LDC in complex with PLP and cadaverine and elucidated the binding mode of cofactor and substrate. Interestingly, compared with the apo-form of *Sr*LDC, the *Sr*LDC in complex with PLP and cadaverine showed a remarkable structural change at the PLP binding site. The PLP binding site of *Sr*LDC contains the highly flexible loops with high b-factors and showed an open-closed conformational change upon the binding of PLP. In fact, *Sr*LDC showed no LDC activity without PLP supplement, and we suggest that highly flexible PLP binding site results in low PLP affinity of *Sr*LDC. In addition, other structurally homologous enzymes also contain the flexible PLP binding site, which indicates that high flexibility at the PLP binding site and low PLP affinity seems to be a common feature of these enzyme family.

## Introduction

Two distinct types of bacterial amino acid decarboxylases have been reported [1]. Biodegradative enzymes such as l-histidine, l-glutamate, and l-lysine decarboxylase are highly induced at low pH and contribute to pH homeostasis [2]. The proton-dependent decarboxylation reaction increases the pH of the growth medium and is used as a defense mechanism against acid stress [3–7]. Biosynthetic enzymes are constitutively expressed regardless of pH variation and include l-ornithine, l-arginine, and l-lysine decarboxylases. These enzymes are responsible for the synthesis of polyamines such as putrescine, spermidine, and cadaverine, and are implicated in a variety of cellular processes [8–10]. In particular, l-lysine decarboxylase (LDC) is an important enzyme for the biosynthesis of cadaverine, which is a crucial platform chemical with many industrial applications.

Cadaverine, also known as 1,5-diaminopentane, is an attractive chemical as a component of polymers such as polyamides and polyurethane [11]. Polyamides have been produced from petrochemicals and are used in a variety of commercial applications. In 2012, the global market was close to USD 22 billion and is estimated to reach USD 27 billion by 2018 [12]. Due to a growing interest in environment-friendly and sustainable resources, it is essential to produce industrially useful chemicals and materials from renewable resources. Bio-based polyamides are good alternatives to previous petrochemical routes in both environmental and economic aspects [13, 14]. Bio-based cadaverine can be obtained from the decarboxylation of l-lysine using LDC, and enzymatic production using external addition of LDC in lysine-rich medium has been proposed [15, 16].

The most attractive approach for a bio-based supply of cadaverine is microbial production using genetically engineered microorganisms such as *Escherichia coli* and *Corynebacterium glutamicum* [17, 18]. Systems metabolic engineering of these strains leads to an improved biosynthetic capacity and a higher cadaverine yield [18–21]. However, two unsolved problems remain in the cadaverine production process. First, as one proton is consumed by the reaction, the pH of the medium increases [22]. Higher pH tends to decrease enzymatic activity, which in turn reduces cadaverine yield. Development of an LDC enzyme that preserves enzymatic activity at high pH is therefore necessary for effective cadaverine production. Second, cadaverine production requires continuous addition of pyridoxal-5-phosphate (PLP) as a cofactor due to the insufficiency of PLP for high level of LDC activity [19]. It has been reported that cadaverine yield increases with the addition of PLP in the medium. In addition, an *in vivo* supply of PLP was engineered, and the resulting additional supply of PLP led to a prolonged LDC reaction and thus to a higher final concentration of cadaverine [23].

In this report, we determined the crystal structures of LDC from *Selenomonas ruminantium* (*Sr*LDC) in different crystallization conditions and observed that high flexibility of the PLP binding site seems to be one of the factors that lead to the low affinity for PLP in *Sr*LDC. By comparing the *Sr*LDC structure with other similar enzymes, the active site flexibility is a common structural feature in this enzyme family.

## Materials and Methods

### Production of *Sr*LDC

The gene coding for lysine decarboxylase from *S*. *ruminantium* (*Sr*LDC, amino acid residues 1–399) was amplified from chromosomal DNA of *S*. *ruminantium* by polymerase chain reaction (PCR) with primers: forward, 5-GCGCG**CATATG**AAAAACTTCCGTTTAAGCGAAAAAG -3, and reverse, 5-GCGCG**CTCGAG**GTGATGGTGATGGTGGTGAACTGCT-3. The PCR product was then subcloned into the pET-22b(+) vector (Life Science Research) with 6x-higtag at the C-terminus. The resulting expression vector pET-22b(+):*Srldc* was transformed into the *E*. *coli* strain BL21(DE3)-T1^R^, which was grown in 1 L of LB medium containing 100 μg/mL ampicillin at 37°C until the OD 600 reached 0.7. After induction with 1.0 mM 1-thio-β-D-galactopyranoside (IPTG), the culture medium was maintained for a further 20 h at 18°C. The culture was then harvested by centrifugation at 4,000 × *g* for 20 min at 4°C. The cell pellet was resuspended in buffer A (40 mM Tris-HCl, pH 8.0) and disrupted by ultrasonication. The cell debris was removed by centrifugation at 13,500 × *g* for 30 min and the lysate was applied to a Ni-NTA agarose column (Qiagen). After washing with buffer A containing 30 mM imidazole, the bound proteins were eluted with 300 mM imidazole in buffer A. Finally, trace amounts of contaminants were removed by size-exclusion chromatography using a Superdex 200 prep-grade column (320 mL, GE Healthcare) equilibrated with buffer A. All purification experiments were performed at 4°C. SDS-polyacrylamide gel electrophoresis analysis of the purified proteins showed a single polypeptide of 44.0 kDa that corresponded to the estimated molecular weight of the *Sr*LDC monomer. The purified protein was concentrated to 65 mg mL^-1^ in 40 mM Tris-HCl, pH 8.0.

### Crystallization of *Sr*LDC

Crystallization of the purified protein was initially performed with commercially available sparse-matrix screens from Rigaku and Molecular Dimensions by using the hanging-drop vapor-diffusion method at 20°C. Each experiment consisted of mixing 1.0 μL of protein solution (65 mg mL^-1^ in 40 mM Tris-HCl, pH 8.0) with 1.0 μL of reservoir solution and equilibrating the drop against 0.5 mL of reservoir solution. The apo-form I crystals of *Sr*LDC were observed in several crystallization screening conditions. After several optimization steps, the best quality crystals appeared in 4 days using a reservoir solution consisting of 28% (w/v) polyethylene glycol (PEG) 400, 0.1 M sodium citrate tribasic-citric acid, pH 6.0, and 0.2 M magnesium chloride at 20°C. The apo-form II crystals of *Sr*LDC were crystallized in the condition containing 1.15 M sodium citrate and 0.1 M sodium cacodylate, pH 5.0. The crystals of *Sr*LDC complexed with PLP/cadaverine were crystallized in the condition of 50% PEG 200, 0.1 M sodium phosphate, pH 4.2, 0.2 M sodium chloride, and 5 mM PLP. We then soaked 10 mM l-lysine into the crystals for 30 min.

### Data collection and structure determination of *Sr*LDC

The crystals were transferred to a cryo-protectant solution composed of the corresponding condition described above and 30% (v/v) glycerol, fished out with a loop larger than the crystals, and flash-frozen by immersion in liquid nitrogen. All data were collected at the 7A beamline of the Pohang Accelerator Laboratory (PAL, Pohang, Korea), using a Quantum 270 CCD detector (ADSC, USA). The crystals of the apo-forms I and II of *Sr*LDC diffracted to 2.0 and 2.9 Å resolutions, respectively. The crystals of *Sr*LDC complexed with PLP/cadaverine crystals diffracted to 2.5 Å resolutions. All data were indexed, integrated, and scaled using the HKL-2000 software package [24]. The apo-form I crystals of *Sr*LDC belonged to the space group *P*4_3_2_1_2 with unit cell parameters *a* = *b* = 105.97 Å, *c* = 73.634 Å, *α* = *β* = *γ* = 90.0°. With one molecule of *Sr*LDC per asymmetric unit, the crystal volume per unit of protein mass was 2.35 Å^3^ Da^-1^, indicating that the solvent content was approximately 47.63% [25]. The apo-form II of *Sr*LDC crystals belonged to the space group *C*2 with unit cell dimensions of *a* = 146.45 Å, *b* = 70.794 Å, *c* = 88.061 Å, *α* = *γ* = 90.0°, *β* = 101.03°. With two molecules per asymmetric unit, the crystal volume per unit of protein mass was 2.54 Å^3^ Da^-1^, which corresponds to a solvent content of 51.68% [25]. The PLP/cadaverine-complexed form of *Sr*LDC crystals belonged to the space group *P*6_3_2_2_ with unit cell dimensions of *a* = *b* = 111.73 Å, *c* = 112.97 Å, *α* = *β* = 90.0°, *γ* = 120.0°. With one molecule per asymmetric unit, the crystal volume per unit of protein mass was 2.31 Å^3^ Da^-1^, and the solvent content was 46.83% [25]. The structure of the apo-form I of *Sr*LDC was solved by molecular replacement with the CCP4 version of MOLREP [26] using the structure of L/ODC from *Vibrio vulnificus* (*Vv*L/ODC, PDB code 2PLK) as a search model. Model building was performed manually using the program WinCoot [27] and refinement was performed with CCP4 refmac5 [28]. The structures of the apo-form II of *Sr*LDC, *Sr*LDC complexed with PLP/cadaverine were determined by molecular replacement using the refined *Sr*LDC structure of apo-form I. Model building and structure refinement of these structures were performed using a procedure similar to that employed for the structure of the apo-form I of *Sr*LDC. Data collection and refinement statistics are summarized in Table 1. Four refined *Sr*LDC structures of apo-from I, apo-form II, and complexed with PLP/cadaverine were deposited in the Protein Data Bank with PDB codes of 5GJN, 5GJM, and 5GJP, respectively.

**Table 1:** Data collection and structural refinement statistics of *Sr*LDC.

	*Sr*LDC		
	Apo I	Apo II	+ PLP/cadaverine
PDB code	5GJN	5GJM	5GJP
**Data collection**			
Space group	*P*4_3_2_1_2	*C*2	*P*6_3_2_2_
Cell dimensions			
*a*, *b*, *c* (Å)	106.0, 106.0, 73.6	146.5, 70.8, 88.1	111.7, 111.7, 113.0
α, β, *γ* (°)	90.0, 90.0, 90.0	90.0, 101.0, 90.0	90.0, 90.0, 120.0
Resolution (Å)	50.0–2.0 (2.03–2.0)*	50.0–2.9 (2.95–2.9)*	50.0–2.5 (2.54–2.5)*
*R*_sym_ or *R*_merge_	7.3 (30.4)	14.5 (34.6)	13.2 (31.7)
*I* / σ*I*	61.0 (10.0)	23.9 (8.5)	89.7 (11.4)
Completeness (%)	99.1 (100.0)	95.2 (94.2)	96.8 (95.1)
Redundancy	11.1 (10.8)	3.5 (3.3)	24.2 (12.9)
**Refinement**			
Resolution (Å)	50.0–2.0	50.0–2.9	50.0–2.5
No. reflections	27192	17748	13670
*R*_work_ / *R*_free_	20.3/24.0	17.7/23.2	19.8/27.9
No. atoms	3107	5913	3034
Protein	2854	5785	2948
Ligand/ion	40	6	48
Water	213	122	38
*B*-factors	44.4	21.2	52.0
Protein	31.7	21.4	51.2
Ligand/ion	59.9	38.7	65.8
Water	48.6	13.1	50.1
B from Wilson plot (Å^2^)	37.5	21.8	43.1
R.m.s. deviations			
Bond lengths (Å)	0.019	0.019	0.019
Bond angles (°)	1.921	1.704	1.636
Ramachandran statistics (%)			
Favored	97.5	97.2	98.6
Allowed	2.5	2.8	1.3
Outliers	0.0	0.0	0.0

^*^The numbers in parentheses are statistics from the highest resolution shell.

### LDC activity assay

To measure the lysine decarboxylase (LDC) activity of the *Sr*LDC proteins, 56.78 μM of purified enzyme was added to 200 μl of reaction mixture containing 0.1 M potassium phosphate, pH 6.0, 50 μM l-lysine, and various concentrations of PLP. The reaction mixtures were incubated at 37°C for 30 sec. The reaction was stopped by heating the solution at 90°C for 5 min. After centrifugation at 13,500 × *g* for 5 min, the remaining l-lysine was detected using the lysine oxidase/peroxidase method. Lysine oxidase converts the remaining l-lysine into 6-amino-2-oxohexanoate, NH_3_, and H_2_O_2_, and the H_2_O_2_ is then reduced by peroxidase using 2,2'-azino-bis(3-ethylbenzothiazoline-6-sulphonic acid) (ABTS). After the LDC reaction, equal volume of 2x lysine oxidase/peroxidase solution (0.1 unit ml^-1^ lysine oxidase, 1 unit ml^-1^ peroxidase, and 3.6 mM ABTS in 0.1 M potassium phosphate buffer, pH 8.0) was added to the LDC reaction mixture. The amount of oxidized ABTS was detected by measuring absorbance at 412 nm. All experiments were performed in triplicate.

### Isothermal titration calorimetry

The binding affinity between *Sr*LDC and PLP was measured using a Nano ITC model (TA Instruments) at 20°C. Protein sample at 100 μM was prepared in 40 mM Tris, pH 8.0. For titrations of *Sr*LDC with PLP, 1.96 μl of 2.5 mM PLP was injected 25 times. Each injection was monitored at 200-second intervals and the protein solution in the ITC reaction cell was stirred at 250 RPM. The heat of cofactor dilution into the buffer was subtracted from the reaction heat. Calculated heat area per injection was used for fitting in the independent binding model for calculation of the thermodynamic parameter (*K*_d_).

## Results and Discussion

### Overall structure of *Sr*LDC

**Fig 1:**
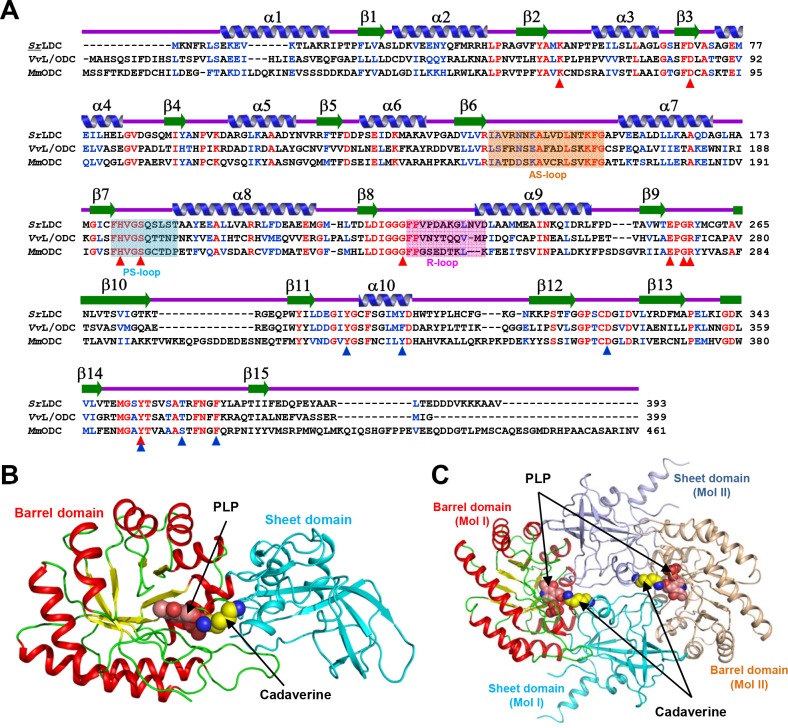
Crystal structure of *Sr*LDC. (A) Amino acid sequence alignment of L/ODCs. Identical and highly conserved residues are presented red and blue colored characters, respectively. Secondary structure elements are drawn based on the *Sr*LDC structure in an apo-form. The AS-loop, PS-loop and R-loop are shown in boxes with orange, cyan and magenta colors, respectively. Residues involved in binding of PLP and substrate are marked with red- and blue-colored triangles, respectively. *Sr*LDC, *Vv*L/ODC, and *Mm*ODC are representatives of lysine decarboxylase from *Selenomonas ruminanitum*, lysine/ornithine decarboxylase from *Vibrio vulnificus*, and ornithine decarboxylase from *Mus musculus*, respectively. (B) Monomeric structure of *Sr*LDC. A monomeric protein is shown as a cartoon diagram. The barrel domain is presented with colors of red and yellow for α-helices and β-strands, and the sheet domain is with a cyan color. The bound PLP and cadaverine are shown as sphere models with salmon and yellow colors, respectively. (C) Dimeric structure of *Sr*LDC. The dimeric structure of *Sr*LDC is shown as a cartoon diagram showing one monomer with a color scheme in (B) and the other monomer in light-orange and light-blue for the barrel domain and the sheet domain, respectively. The bound PLP and cadaverine are shown as sphere models with salmon and yellow colors, respectively.

To reveal the molecular mechanism of *Sr*LDC, we determined its crystal structure at 2.0 Å resolution (Table 1). The asymmetric unit contains one *Sr*LDC molecule, and the dimeric structure of the protein could be easily generated by applying crystallographic *P*4_3_2_1_2 symmetry. *Sr*LDC shows an overall fold similar to that of other constitutively expressed ornithine decarboxylases such as *Vibrio vulnificus* lysine/ornithine decarboxylase (*Vv*L/ODC, PDB code 2PLK), *Mus musculus* ODC (*Mm*ODC, PDB code 7ODC), and *Trypanosoma brucei* ODC (*Tb*ODC, PDB code 1F3T) (Fig 1A). The monomeric form of *Sr*LDC consists of two distinct domains, a PLP-binding barrel domain, and a sheet domain. The barrel domain (Leu27-Cys261) is composed of eight α-helices (α2-α9) and eight parallel β-strands (β2-β9) in an alternating pattern (Fig 1B). The eight α-helices wrap the eight-stranded β-sheet core that serves as a cofactor binding site. The sheet domain (Met1-Ser26, Gly262-Val393) is formed by a seven-stranded anti-parallel β-sheet (β1, β10-β15) that is surrounded by three short α-helices (Fig 1B). This domain contributes mainly to dimerization. The *Sr*LDC homodimer is formed by a head-to-tail contact between the barrel domain of one monomer and the sheet domain of the other monomer (Fig 1C). PISA software [29] calculated that a 2,474.4 Å^2^ area of solvent-accessible interface per monomer is buried, and the percentage of participating residues is 19.7%. Two identical active sites lie at the dimer interface and both active sites contain residues from each monomer.

### Cofactor and substrate binding mode of *Sr*LDC

**Fig 2:**
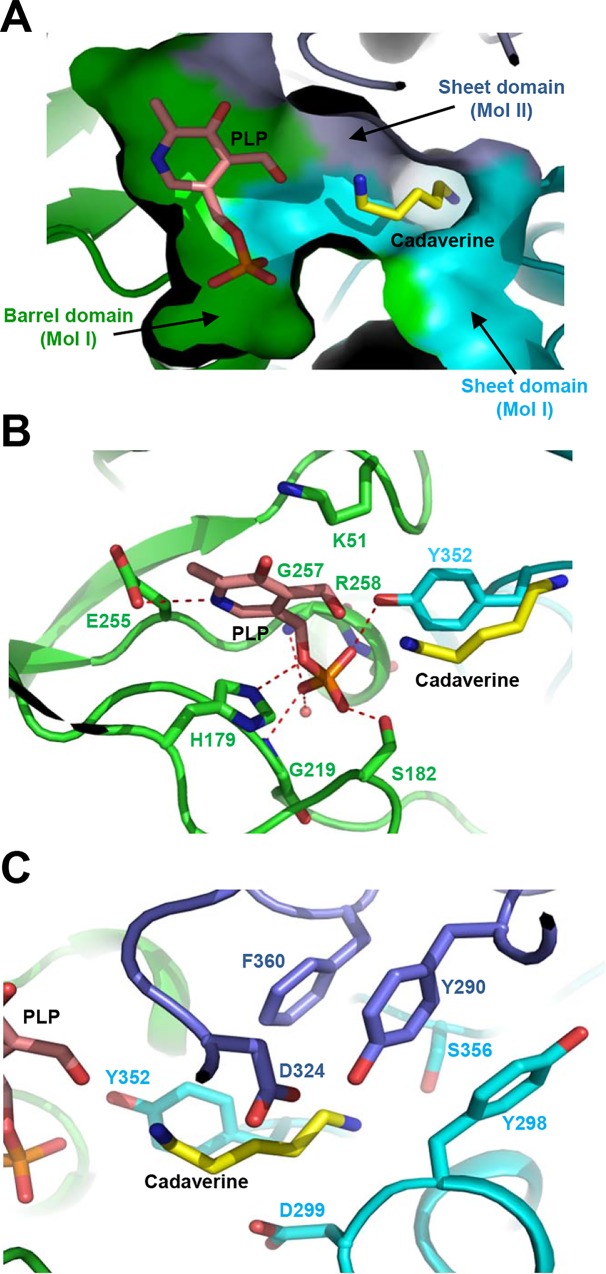
Cofactor and substrate binding mode of *Sr*LDC. (A) A surface model of the active site of *Sr*LDC. The *Sr*LDC structure complexed with PLP/cadaverine is presented with a surface model with colors of green, cyan, and light-blue for the barrel domain and the sheet domain of one monomer and the sheet domain of the other monomer, respectively. The bound PLP and cadaverine are shown as stick models with salmon and yellow colors, respectively. (B) PLP binding mode of *Sr*LDC. The *Sr*LDC structure complexed with PLP/cadaverine is presented with a cartoon diagram with the same color scheme as in (A). The residues involved in the PLP binding are shown as stick models and labeled. The hydrogen bonds involved in the PLP binding are shown as red-colored dotted lines. The bound PLP and cadaverine are shown as stick models with salmon and yellow colors, respectively. (C) Substrate binding mode of *Sr*LDC. The *Sr*LDC structure complexed with PLP/cadaverine and residues involved in the substrate binding are presented as (B).

To understand the cofactor and substrate binding modes of *Sr*LDC, we determined the crystal structure of this enzyme complexed with PLP and cadaverine at 2.5 Å resolution. Unlike the apo-form, the *Sr*LDC structure complexed with PLP/cadaverine belongs to the *P*6_3_2_2_ space group (Table 1). Although we soaked the crystals of *Sr*LDC complexed with PLP in l-lysine, a cadaverine molecule was observed instead. We suspect that l-lysine was converted into cadaverine during the soaking process. The PLP cofactor binds mainly to the pocket formed at the barrel domain (Fig 2A), and the catalytic residue Lys51 interacts with the aldehyde group of the pyridoxal ring. The pyridine ring is stabilized by hydrogen bond between N1 of the ring and the acidic residue Glu255 (Fig 2B). The phosphate moiety of PLP is mainly stabilized by strong hydrogen bonds with the side-chains of His179, Ser182, and Tyr352, and the main-chains of Gly219, Gly257, and Arg258 are also involved in the stabilization of the moiety (Fig 2B). A shallow substrate-binding hole is formed at the interface between the sheet domains of two monomers (Fig 2A). The ε-amino group of the cadaverine product seems to be stabilized by hydroxyl groups of residues Tyr290 and Ser356. Hydrophobic residues such as Tyr298 and Phe360 provide hydrophobicity for the stabilization of five methylene groups of cadaverine. Asp299 also aids the stabilization of hydrophobic parts of cadaverine. Residues Asp324 and Tyr352 are located in the vicinity of the α-amino group of cadaverine (Fig 2C).

### High flexibility at the active site of *Sr*LDC

**Fig 3:**
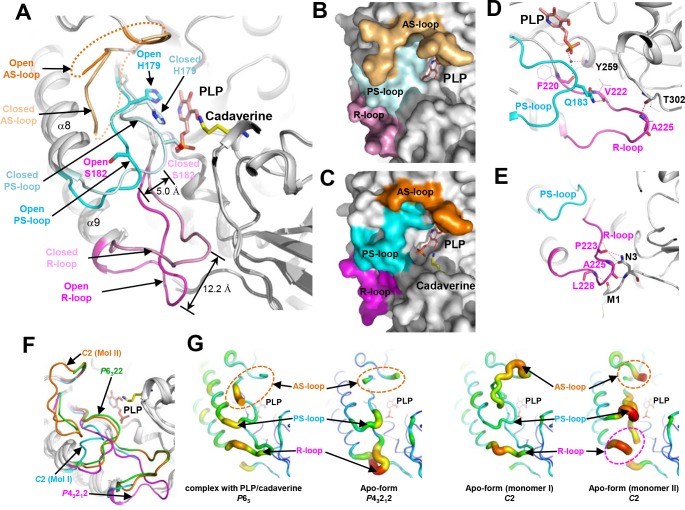
Highly flexible active site of *Sr*LDC. (A) Open and closed conformational changes of the active site of *Sr*LDC. The *Sr*LDC structures in the apo-form and complexed with PLP/cadaverine are superimposed and presented with cartoon diagrams. The AS-loop, the PS-loop, and the R-loop in the open and closed conformations are distinguished with different colors and labeled. The bound PLP and cadaverine are shown as stick models with salmon and yellow colors, respectively. (B) (C) Surface models of the open and closed conformations. The AS-loop, the PS-loop, and the R-loop in the closed (B) and open (C) conformations are presented with surface models with the same color scheme as in (A). (D) (E) Stabilization mode of the PS-loop and the R-loop. The PS-loop and the R-loop in the closed (D) and open (E) conformations are presented with cartoon diagrams with the same color scheme as in (A). Side-chains involved in the stabilization of the PS-loop and the R-loop are shown as stick models. Main-chains involved in the stabilization of the PS-loop and the R-loop are shown as stick models and their side-chains are as line models. Hydrogen bonds are presented as red-colored dotted lines. (F) Superimposition of *Sr*LDC structures in three different crystal forms. Four monomeric *Sr*LDC structures in three different crystal forms are superimposed and presented as cartoon diagrams. The AS-loop, PS-loop, and the R-loop of four monomers are distinguished with different colors and labeled. (G) B-factor presentation of *Sr*LDC structures in three different crystal forms. The crystal forms are labeled at the bottom of the figures. The AS-loop, PS-loop, and the R-loop are labeled.

Importantly, we observed a large conformational change in *Sr*LDC upon binding of the PLP cofactor. When we superimpose the structure of the apo-form of *Sr*LDC in *P*4_3_2_1_2 space group with that of *Sr*LDC complexed with PLP/cadaverine, the overall shape of the two structures is almost the same, with an R.M.S.D. value of 0.72 Å. However, remarkable structural differences are observed at the active site (Fig 3A). Interestingly, in the structure of the apo-form of *Sr*LDC, the connecting loop between β-7 and α-8 (Phe178-Thr187) moved away from the PLP binding site by a distance of 5.0 Å, compared with the structure of *Sr*LDC complexed with PLP/cadaverine (Fig 3A). The loop is heavily involved in the stabilization of PLP by forming hydrogen bonds between residues His179 and Ser182 and the phosphate moiety of PLP (Fig 3A). Because the conformational change disrupts these hydrogen bonds between the loop and the phosphate moiety of PLP, it might severely impair binding of the cofactor to the protein. Hereafter, the loop will be referred to as a PLP stabilization loop (PS-loop). Also interestingly, the conformational change of the PS-loop subsequently causes a more dramatic conformational change in the loop connecting β-8 and α-9 (Phe220-Asp231). The loop, located near the PLP binding site in the structure of *Sr*LDC complexed with PLP/cadaverine, moved away from the PLP binding site by a distance of 12.2 Å in the structure of the apo-form (Fig 3A). These structural observations clearly show that *Sr*LDC has an open conformation in the apo-form and a closed conformation in the complexed form (Fig 3B and 3C). The stabilization modes of the loop are also quite different from each other. In *Sr*LDC complexed with PLP/cadaverine, the main-chain of Phe220 forms a water-mediated hydrogen bond network with the main-chain of Tyr259 and phosphate moiety of PLP. The main-chain of Ala225 forms a direct hydrogen bond with the main-chain of Thr302. The loop also interacts with the PS-loop by a hydrogen bond between the side-chain of Q183 and the main-chain of Val222 (Fig 3D). In contrast, in the apo-form of *Sr*LDC, the loop is stabilized by the N-terminal region instead: the main-chains of Met1 and Leu228 form a hydrogen bond with each other, and the main-chains of Pro223 and Ala225 form hydrogen bonds with both the side- and the main-chains of Asn3 (Fig 3E). The loop conformation seems to directly influence the PS-loop conformation, and we will refer to this loop as regulatory loop (R-loop). An additional loop worth mentioning is the one connecting β-6 and α-7 (Ile137-Gly153). This loop is known to be disordered or highly flexible in this family of enzymes, and to undergo conformational change controlling the substrate’s access to the active site [30, 31]. The loop is also disordered both in the apo-form of *Sr*LDC and in the form complexed with PLP/cadaverine (Fig 3A). Considering the function of the loop, we will refer to it as active site loop (AS-loop).

The observed conformational changes of the three loops, PS-, R-, and AS-loop, lead us to propose that *Sr*LDC undergoes large open-closed conformational changes upon binding of the PLP cofactor and/or the lysine substrate. However, the space groups of the *Sr*LDC structures in the apo-form (*P*4_3_2_1_2) and in the form complexed with PLP/cadaverine (*P*6_3_2_2_) are different from each other (Table 1). Because different crystal packing can influence the local conformations of proteins, the question of whether the observed open-closed conformational changes are physiologically relevant remains. To investigate the conformational changes at the active site of *Sr*LDC, we determined its crystal structure in the apo-form with a *C*2 space group at 2.9 Å resolution (Table 1). Unlike the two crystal forms described above, the structure contains a dimer in an asymmetric unit. Interestingly, two *Sr*LDC monomers in the asymmetric unit exhibit completely different conformations at the active site, although no cofactor or substrate is bound in either monomer. In one monomer, the PS-loop is in the open-conformation and the R-loop is disordered (Fig 3F). In contrast, the other monomer contains both the PS-loop and the R-loop in the closed-conformation observed in the structure of *Sr*LDC complexed with PLP/cadaverine. Moreover, the entire AS-loop is in an ordered-conformation with a clear electron density map in this region (Fig 3F). These observations indicate that the open-closed conformation of *Sr*LDC is caused mainly by the different crystal packings rather than by the binding of the PLP cofactor and/or the lysine substrate (S1 Fig). In fact, the PS-loop, the R-loop, and the AS-loop all show a much higher b-factor compared with the rest of the protein (Fig 3G), indicating that these loop regions are highly flexible. Taken together, the results allow us to conclude that the active site of *Sr*LDC has an extremely high flexibility.

### Low PLP affinity in *Sr*LDC

**Fig 4:**
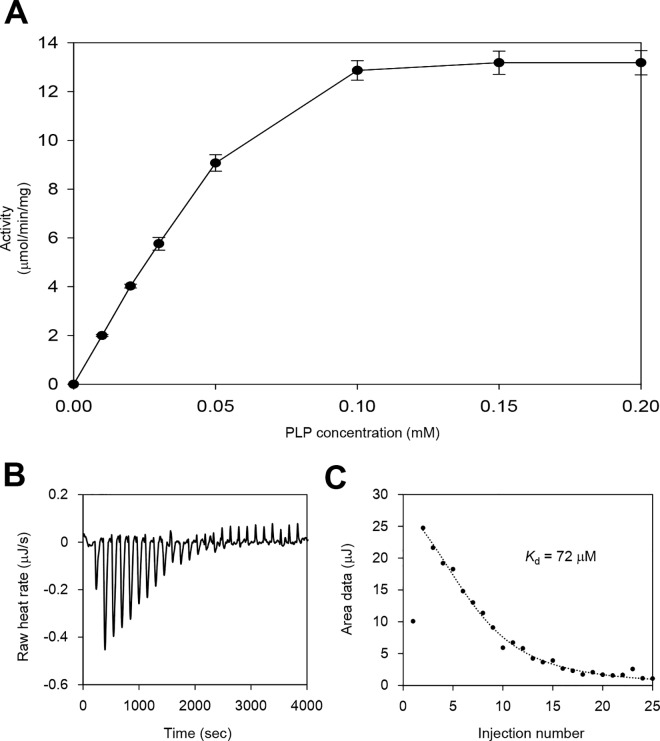
LDC Activity of *Sr*LDC. (A) LDC activity of *Sr*LDC at various concentrations of PLP. LDC activity of *Sr*LDC was measured with various concentrations of PLP (0, 0.01, 0.02, 0.03, 0.05, 0.1, 0.15, and 0.2 mM). All experiments were performed in triplicates. (B) (C) Raw (B) and fitted (C) data of Isothermal titration calorimetry of *Sr*LDC. The binding interactions between *Sr*LDC and PLP were carried out using isothermal titration calorimetry (Nano ITC model, TA Instruments).

As described above, *Sr*LDC exhibits highly flexible active site. We then tested the PLP requirements of *Sr*LDC by measuring the LDC activity of the recombinant protein in various concentrations of PLP. As expected, the recombinant *Sr*LDC protein showed no noticeable activity in the reaction mixture without PLP supplement. When various concentrations of PLP were added to the reaction mixture, activity of the recombinant *Sr*LDC was detected and reached its maximum at 0.1 mM or higher concentrations of PLP (Fig 4A). These results indicate that the PLP cofactor was detached from the recombinant *Sr*LDC protein during the purification procedure, and the enzyme thus showed no activity without PLP supplement. To investigate if requirement of the additional PLP supplement for LDC activity is due to the low affinity of *Sr*LDC for PLP, we measured the K_d_ value of the protein for PLP using isothermal titration calorimetry. The result showed that *Sr*LDC has a somewhat high K_d_ value of 72 μM (Fig 4B and 4C). Interestingly, other PLP-dependent enzymes such as tryptophan synthase and tyrosine phenol lyase exhibited much higher PLP affinity than *Sr*LDC with K_d_ values of 0.9 μM [32]and 2.0 μM [33]. Based on these results and the structural observations described above, we propose that highly flexible active site might contribute to the low affinity for the PLP cofactor in *Sr*LDC.

### Flexible active sites of other L/ODCs

**Fig 5:**
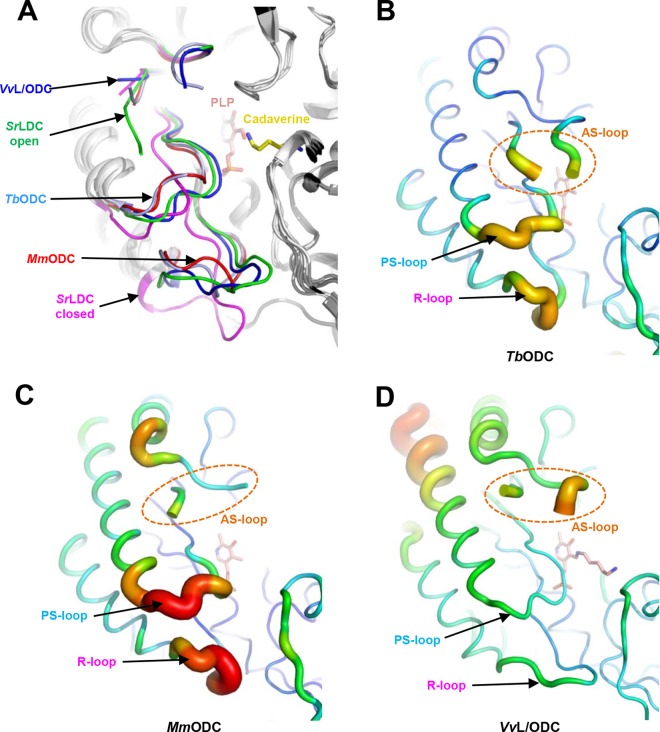
Flexible active sites of other L/ODCs. (A) Superimposition of L/ODC structures. Structures of the open and closed conformations of *Sr*LDC and those of *Tb*ODC, *Mm*ODC, and *Vv*L/ODC are superimposed. The AS-loop, PS-loop and the R-loop of these structures are distinguished with different colors and labeled. (B) (C) (D) B-factor presentation of the structures of *Tb*ODC (B), *Mm*ODC (C), and *Vv*L/ODC (D). The AS-loop, PS-loop and the R-loop of these structures are labeled.

We then investigated the active site flexibility of other structurally homologous enzymes. When we superimposed the *Sr*LDC structure with the PLP-complexed structures of *Vv*L/ODC, *Mm*ODC, and *Tb*ODC, the overall structures were quite similar to each other, with R.M.S.D. values of 1.2, 1.4, and 1.4 Å, respectively. Although all three PLP-complexed L/ODC structures show closed conformations similar to that observed in the structure of *Sr*LDC complexed with PLP/cadaverine, the PS-loop and R-loop conformations are somewhat different in each structure. The PS-loops of *Mm*L/ODC and *Tb*ODC are positioned slightly away from the PLP binding site compared with that of *Sr*LDC (Fig 5A). In the *Mm*ODC structure in particular, this conformational difference resulted in a water-mediated hydrogen bonding between Ser200 and the phosphate moiety instead of a direct hydrogen bond found in other proteins (S2 Fig). More pronounced differences were observed in R-loops. Although the R-loop of *Vv*L/ODC shows a conformation quite similar to that of the closed form of *Sr*LDC, the R-loops of *Mm*O/LDC and *Tb*ODC are in an intermediate position between the open and the closed conformations of *Sr*LDC (Fig 5A). Most importantly, the AS-, PS- and R-loops of these structures exhibit a high b-factor, similar to those observed in *Sr*LDC (Fig 5B, 5C and 5D). One exception is *Vv*L/ODC, which shows a relatively low b-factor compared to other structures, however, this difference seems to be derived from the tight crystal packing of the structure (S3 Fig). In addition, it has been reported that PLP was added to the reaction mixture during the enzymatic activity assays of these enzymes. Based on these results, we suggest that a flexible active site is not confined only to *Sr*LDC, but rather is a common structural feature of this enzyme family.

## Conclusion

PLP is a versatile cofactor and is utilized in a variety of reactions [34]. In particular, PLP-dependent enzymes are involved in the production of amino acids and amine-containing compounds by means of their decarboxylation and transamination activities. However, due to the low PLP binding affinity, several PLP-dependent enzymes require additional supplements of PLP to maintain their activities [35]. For this reason, PLP has been one of the critical control factors for the production of industrially important materials. Many attempts have been also made to minimize the additional cost of a continuous supply of PLP in the production of cadaverine. Recently, a *de novo* PLP biosynthetic pathway was introduced into the cadaverine-producing strains to increase the cellular level of PLP [23]. The structural studies in this report provides structural basis for low PLP affinity of LDCs, and might be utilized for future protein engineering to enhance the affinity for PLP in this family enzymes.

## Supporting Information

S1 Fig Different contacts of *Sr*LDC with neighboring molecules in different crystal forms. One *Sr*LDC structure is shown as a cartoon diagram. The AS-loop, PS-loop and the R-loop of *Sr*LDC are colored orange, cyan, and magenta, respectively. The molecules near *Sr*LDC are shown as surface models with different colors.
(TIF)

S2 Fig Mode of hydrogen bonding between a serine residue on the PS-loop and PLP. The Ser182 residue of *Sr*LDC and the corresponding residues in other O/LDCs are shown as stick models and labeled. Hydrogen bonds between the serine residue and the phosphate moiety of PLP are shown as red-colored dotted lines.
(TIF)

S3 Fig Contacts of *Vv*L/ODC with neighboring molecules. One *Vv*L/ODC structure is shown as a cartoon diagram. The AS-loop, the PS-loop and the R-loop of *Vv*L/ODC are colored orange, cyan, and magenta, respectively. The molecules near *Vv*L/ODC are shown as surface models with different colors.
(TIF)
