# Supplementary figures and images for: Crystal Structure and Pyridoxal 5-Phosphate Binding Property of Lysine Decarboxylase from Selenomonas ruminantium
Source: PLoS One. 2016 Nov 18;11(11):e0166667. doi: 10.1371/journal.pone.0166667 (PMC5115768; doi:10.1371/journal.pone.0166667)

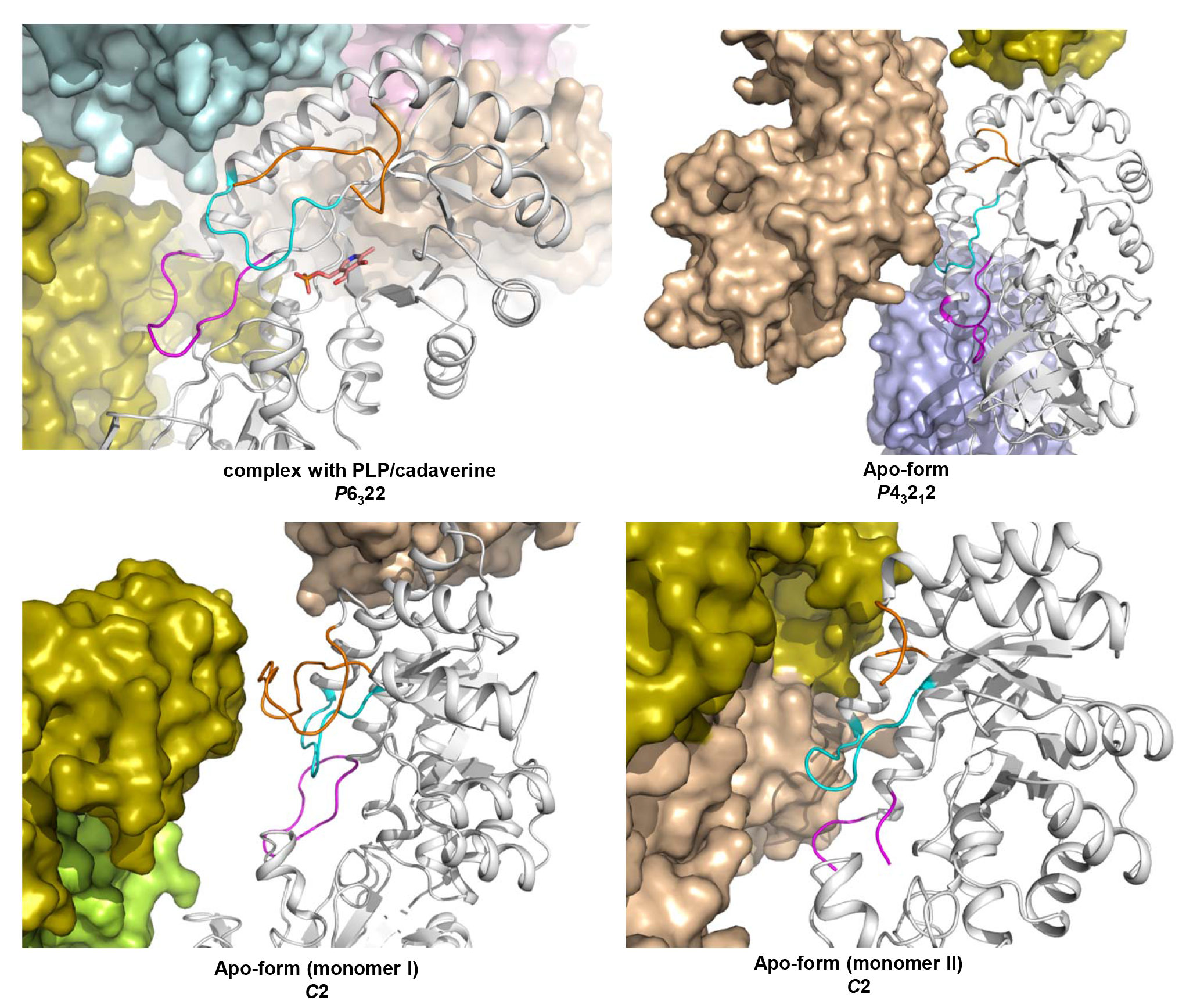

Supplement: S1 Fig — One SrLDC structure is shown as a cartoon diagram. The AS-loop, PS-loop and the R-loop of SrLDC are colored orange, cyan, and magenta, respectively. The molecules near SrLDC are shown as surface models with different colors. (TIF) [file pone.0166667.s001.tif]

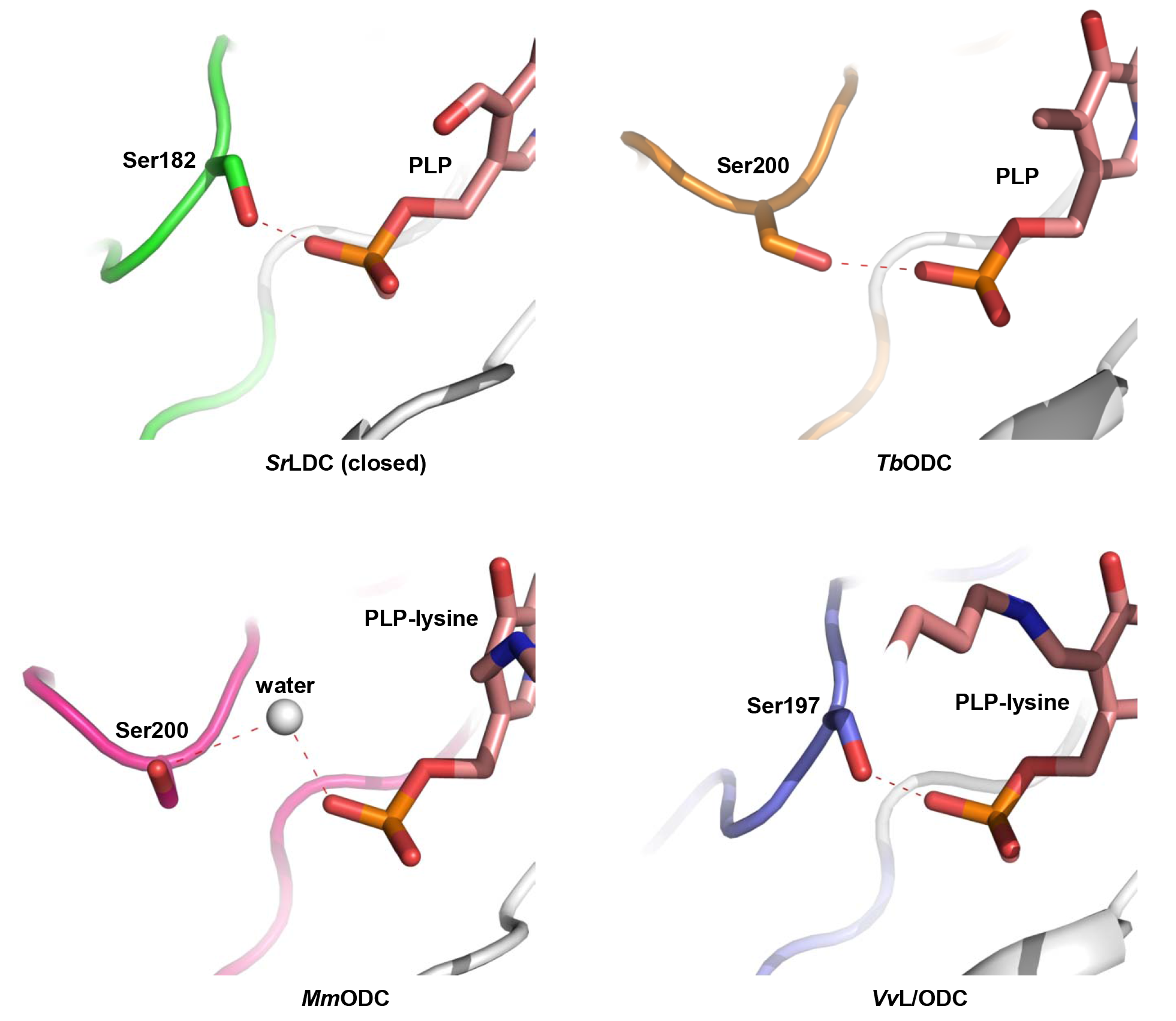

Supplement: S2 Fig — The Ser182 residue of SrLDC and the corresponding residues in other O/LDCs are shown as stick models and labeled. Hydrogen bonds between the serine residue and the phosphate moiety of PLP are shown as red-colored dotted lines. (TIF) [file pone.0166667.s002.tif]

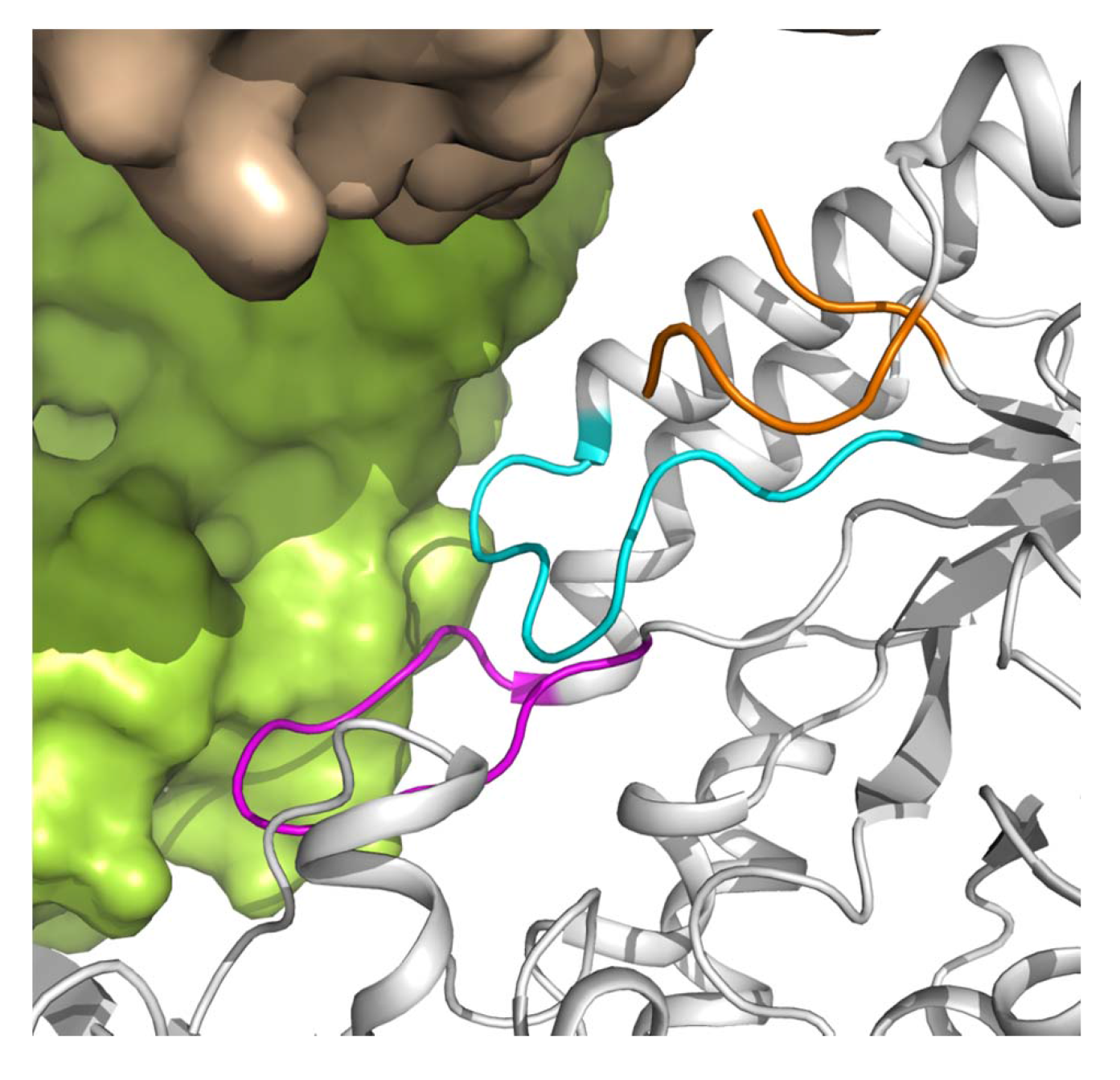

Supplement: S3 Fig — One VvL/ODC structure is shown as a cartoon diagram. The AS-loop, the PS-loop and the R-loop of VvL/ODC are colored orange, cyan, and magenta, respectively. The molecules near VvL/ODC are shown as surface models with different colors. (TIF) [file pone.0166667.s003.tif]
